# Antibodies against *Toxoplasma gondii* positive in serum and aqueous humor to diagnose clinically suspected ocular toxoplasmosis: A case report

**DOI:** 10.1097/MD.0000000000030956

**Published:** 2022-10-07

**Authors:** Lixia Niu, Sufang Wang, Yunyun Li, Jun Liu

**Affiliations:** a Department of Ophthalmology, Changzhi People’s Hospital, Changzhi, China.

**Keywords:** aqueous humor, IgG antibody, ocular toxoplasmosis, serology, toxoplasma

## Abstract

Previously, diagnosis of ocular toxoplasmosis is based on clinical symptoms and *Toxoplasma* serology. Checking serological indicators often cannot reflect the real intraocular situation, and may even mislead clinicians to make wrong judgments.

**Patient concerns:**

A 38-year-old male patient visited our ophthalmology clinic with a chief complaint of decreased vision for about 5 days in his right eye.

**Diagnosis:**

Aqueous humor sample analysis found Toxoplasma DNA detectable, and Toxoplasma immunoglobulin G (IgG) and immunoglobulin M (IgM) to be positive. His serum Toxoplasma IgG was also positive (10.04 IU/mL; reference range: 0 to 7.2 IU/mL). Therefore, the final diagnose was ocular toxoplasmosis involving his right eye.

**Interventions:**

Oral prednisone 60 mg/day and azithromycin 0.25 g/day were started. Oral antibiotic treatment for toxoplasma was continued for 4 weeks, and prednisone followed by weekly stepwise tapering in steps of 10 mg/day.

**Outcomes:**

The BCVA and fundus of right eye remained stable after treatment at follow-up.

**Conclusions:**

This article reported a case of ocular *Toxoplasma gondii* infection diagnosis by serum and aqueous humor antibody tests. We provide some additional information on the *T gondii* infection diagnosis.

## 1. Introduction

*Toxoplasma gondii* is an intracellular parasite with cats or other pets as their final host and humans as well as other mammals as intermediate hosts. It causes systemic infection of the intermediate host.^[1]^ *Toxoplasma* infection is divided into congenital and acquired infections, and is an extremely important cause of uveitis, which often causes irreversible damage to vision.^[2,3]^ Toxoplasmic chorioretinitis usually presents as localized milky white exudative foci retinal choroiditis and markedly cloudy vitreous. In most cases, the pathogenic examination of *Toxoplasma* is difficult, and the positive rate is relative low, therefore, the diagnosis of *Toxoplasma* is mostly based on serological examination.^[4]^ However, serological tests alone cannot confirm the diagnosis, particularly in patients with suspected ocular *Toxoplasmosis*, thus, the detection of serum anti-toxoplasma antibodies can only be used as a diagnostic reference, and more importantly, the detection of *Toxoplasma* antibodies in intraocular fluid should be performed. Here, we reported a case with ocular *toxoplasmosis* diagnosed by serum and aqueous humor sample antibody tests. This study was approved by the Ethics Committee of Changzhi People’s Hospital. All procedures conformed to the Declaration of Helsinki, and informed consent was obtained from the patient.

## 2. Case presentation

**Figure 1.**
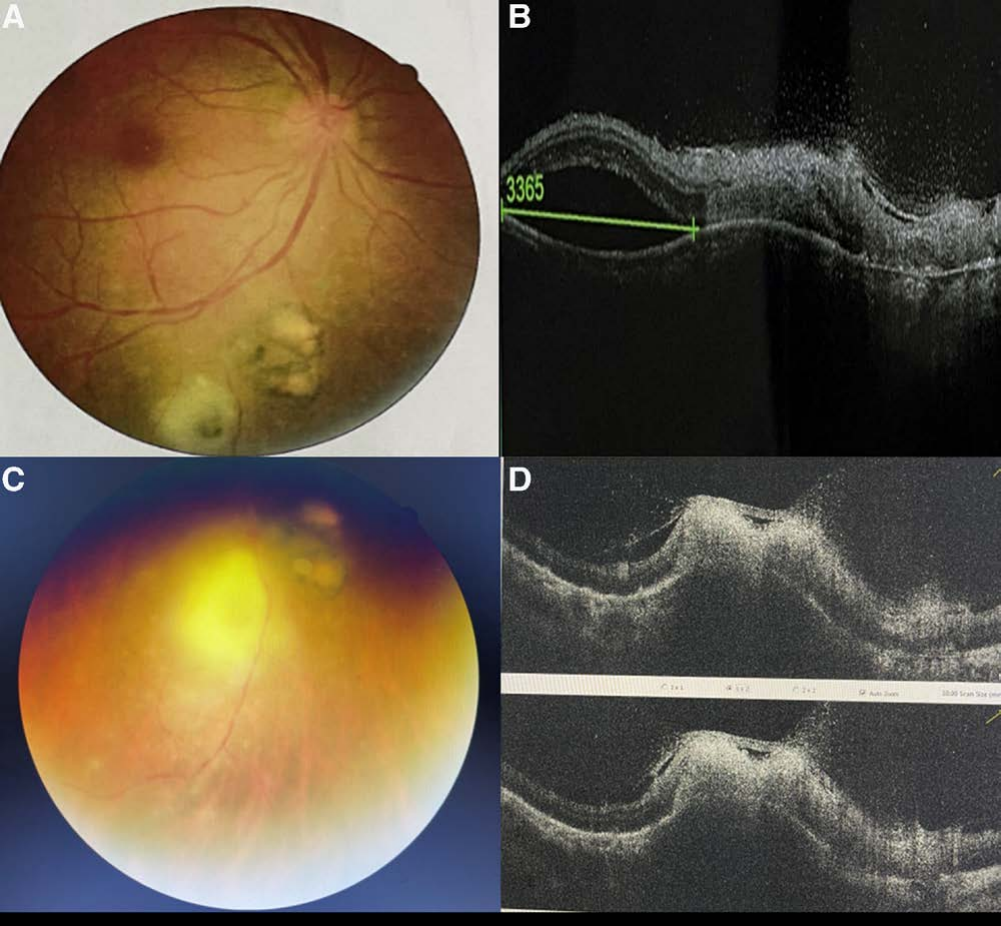
Fundus photograph and SD-OCT image of the patient. (A) Fundus photograph at presentation; (B) SD-OCT image at presentation; (C) Fundus photograph after 3 days presentation; (D) SD-OCT image after 3 days presentation.

**Figure 2.**
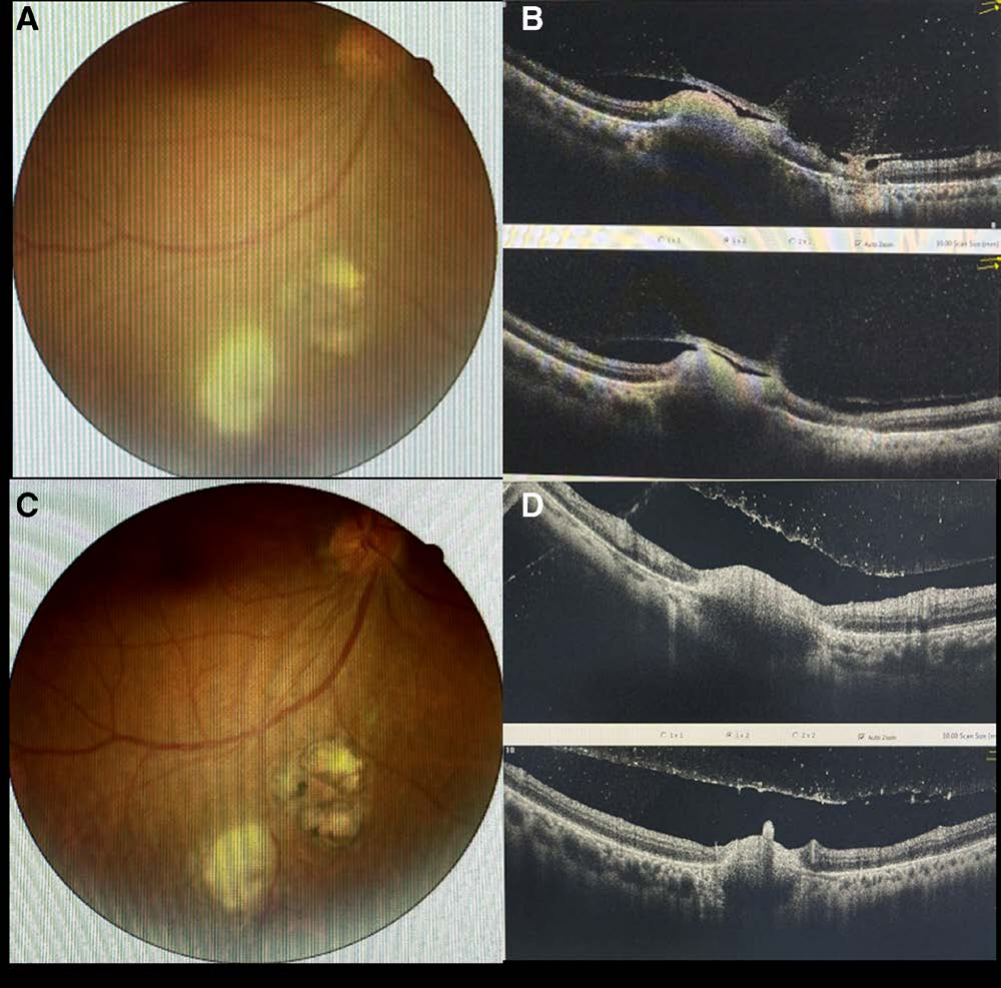
Fundus photograph and SD-OCT image of the patient. (A) Fundus photograph after 7 days medication; (B) SD-OCT image after 7 days medication; (C) Fundus photograph after 3 weeks medication; (D) SD-OCT image after 3 weeks medication.

A 38-year old male patient visited our ophthalmology clinic with a chief complaint of decreased vision for about five days in his right eye. He had no history of any systemic illness such as diabetes, hypertension, trauma or surgery. He denied exposure to animals such as cats and dogs, eating undercooked meat or drinking unfiltered water. The best-corrected visual acuity (BCVA) was 18/20 in his right eye and 20/20 in his left eye, respectively. The intraocular pressure (IOP) was normal in both eyes. A little punctate gray-white keratopigmentation was found under the cornea, positive atrial flashes, and light vitreous turbidity in his right eye. The fundus examination of his right eye showed a round lesion with two optic disc sizes, having clear boundary and pigmentation in the inferior temporal vascular arch of the retina. Below it, there was a yellow-white round retinochoroidal lesion, about 1.5 optic disc sizes, with poorly defined borders and a small patch of pigmentation in the center (Fig. 1A). Spectral-domain optical coherence tomography (SD-OCT) confirmed mild edema of the neuroepithelial layer in active lesions, with small-scale serous detachment, unclear interlayer structure of neuroepithelial layer in old lesions, atrophy and thinning, and slightly stronger reflex (Fig. 1B). A preliminary diagnosis of parasitic infection was given based on the choroidoretinopathy in his right eye. In order to confirm our diagnosis, 0.5 mL of aqueous humor and serum sample were collected and prepared for parasite testing. Unfortunately, the patient’s condition progressed rapidly. His BCVA dropped to 20/60, the vitreous opacity was significantly increased, and the scope of the active lesions in the right eye fundus examination was slightly enlarged, with a size of about two optic disc sizes, then, the boundary of lesions were blurred, and the milky yellow lesions deepened (Fig. 1C). SD-OCT showed thickening of the entire neuroepithelial layer at the lesion, enhanced reflexes, and disappearance of the structure (Fig. 1D). Aqueous humor sample analysis found *Toxoplasma* DNA detectable, and *Toxoplasma* immunoglobulin G (IgG) and immunoglobulin M (IgM) to be positive. His serum Toxoplasma IgG was also positive (10.04 IU/mL; reference range: 0–7.2 IU/mL). In addition, the laboratory tests showed that the patient was negative for anti-HIV, anti-Herpes, anti-Varicella and anti-CMV antibodies and VDRL-RPR. The Goldmann-Witmer coefficient value of *T gondii* was 12 (reference range 0–2). Therefore, the final diagnose was ocular *Toxoplasmosis* involving his right eye. Oral prednisone 60 mg/day and azithromycin 0.25 g/day were started. Oral antibiotic treatment for *Toxoplasma* was continued for 4 weeks, and prednisone followed by weekly stepwise tapering in steps of 10 mg/day. In the follow-up session after 7 days, the BCVA of the right eye was 18/20, IOP was normal in both eyes, and no cells were observed in the anterior chamber of right eye. The fundus examination of his right eye showed yellow-white round retinochoroidal lesions with the size of of 1.5 optic discs, and SD-OCT showed a similar lesion as previous (Fig. 2A and B). Three weeks after the medication, the patient’s BCVA was still 18/20, fundus examination revealed reduction changes of the pigmented chorioretinal scars and yellow-white retinochoroidal lesions (1 optic disc size, Fig. 2C). A repeat SD-OCT showed a reduction in retina thickness (Fig. 2D).

**Figure 3.**
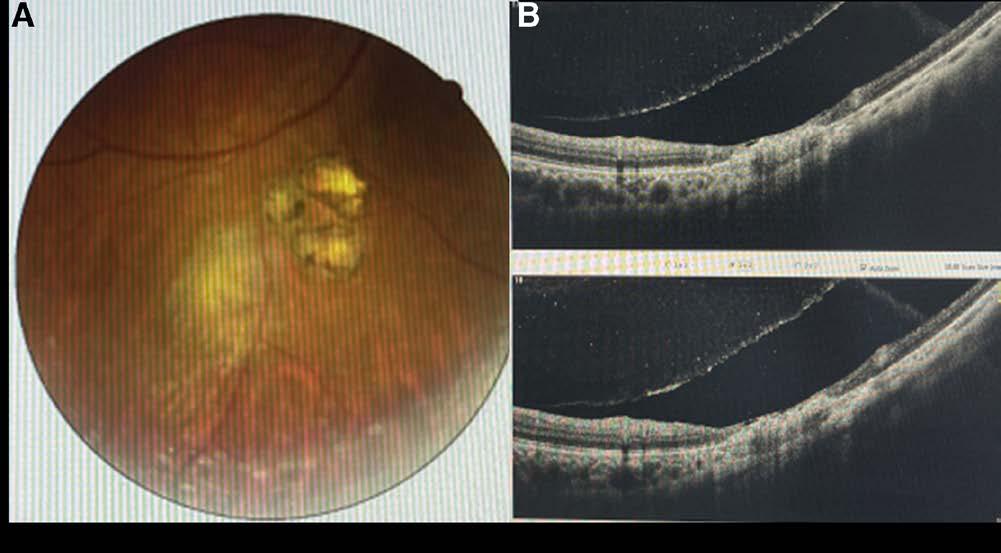
Fundus photograph and SD-OCT image of the patient. (A) Fundus photograph after 3 months medication; (B) SD-OCT image after 3 months medication.

Three months after the medication, the patient’s BCVA increased to 20/20. His IOP was still normal. The lens and vitreous were clear. Fundus examination revealed obvious the pigmented chorioretinal scars and yellow-white retinochoroidal lesions (1 optic disc size, Fig. 3A). A SD-OCT showed no edema of the neuroepithelial layer at the lesion subsided, retina atrophy and thinning (Fig. 3B).

## 3. Discussion

In this case, we experienced a case of acute chorioretinitis according to anterior and fundus examinations. In ruling out infections, ocular *T gondii* infection was made due to the Toxoplasma IgG and IgM tests to be positive, which indicated a “distant” infection. From these two findings, we ruled out recent infection. Notably, travel history was not significant, and this patient denied exposure to cats, dogs or other pets. However, it should be noted that *Toxoplasma* antibody positive is still a multiplex profile consisting of reinfection, reactivation, and latent infection.^[5]^ Determine the infection profile of toxoplasma might pose a diagnostic challenge in some cases. Therefore, careful diagnosis considering both ophthalmic examinations and serology of such patients are needed.

In most cases with ocular *Toxoplasmosis*, a classic fundus examination showed a chorioretinitis, characterized by being an atypical inflammatory yellow-white, round, single lesion of approximately an optic disc in diameter.^[6,7]^ The serology test detected anti-*T gondii* IgG positive. Since the serum antibody level of patients with *T gondii* retinochoroiditis is generally low, and the level of antibody has no significant correlation with the severity of fundus lesions,^[8]^ therefore, any serum anti-toxoplasma antibody positive regardless of the titer, as long as it is combined with typical fundus lesions diagnosis.

With the development of intraocular fluid detection technology, the detection of anterior aqueous humor and vitreous provides a new diagnostic basis for unexplained infectious intraocular diseases.^[9]^ In this case, in order to clarify our hypothetical diagnosis, we promptly tested the patient’s anterior aqueous humor, which was positive for *T gondii* antibodies, probably in the early stage of the disease. Then, the patient underwent emerging treatment and brought the condition under control.

The treatment of *Toxoplasmosis* depends on the location, nature and severity of the lesion. Old lesions generally do not require treatment. The treatment method in the acute phase is sufficient anti-toxoplasma drugs combined with glucocorticoids. The traditional treatment is pyrimethamine combined with sulfa drugs, which has serious side effects. In recent years, there have been many studies on the application of azithromycin in the treatment of *Toxoplasmosis*.^[10]^ After the combination therapy with antibiotic and glucocorticoids (prednisone) three months, the patient`s right eye was still in stable condition with the absence of active inflammation.

In sum, we reported a case of ocular *T gondii* infection diagnosis by serum and aqueous humor antibody tests. We hope to provide some additional information on the *T gondii* infection diagnosis.

## Author contributions

JL performed the patient care and diagnosis. LXN, and YYL analyzed the laboratory diagnosis. JL, SFW and LXN wrote the first draft. All authors contributed to the article and approved the submitted version.

**Conceptualization:** Lixia Niu, Yunyun Li, Jun Liu.

**Data curation:** Lixia Niu, Jun Liu.

**Investigation:** Sufang Wang.

**Methodology:** Sufang Wang, Yunyun Li.

**Software:** Yunyun Li.

**Supervision:** Jun Liu.

**Writing – original draft:** Lixia Niu, Sufang Wang, Yunyun Li, Jun Liu.

**Writing – review & editing:** Lixia Niu, Sufang Wang, Yunyun Li, Jun Liu.
